# (*Z*)-3-(9-Anthr­yl)-1-(4-bromo­phen­yl)-2-(4-nitro-1*H*-imidazol-1-yl)prop-2-en-1-one

**DOI:** 10.1107/S1600536809018352

**Published:** 2009-05-23

**Authors:** Yi-Hui Lu, Guang-Zhou Wang, Cheng-He Zhou, Yi-Yi Zhang

**Affiliations:** a School of Chemistry and Chemical Engineering, Southwest University, Chongqing 400715, People’s Republic of China

## Abstract

In the title mol­ecule, C_26_H_16_BrN_3_O_3_, the anthracene and benzene mean planes make dihedral angles of 63.79 (2) and 14.67 (2)°, respectively, with the plane of the imidazole ring. In the crystal structure, weak inter­molecular C—H⋯O hydrogen bonds link mol­ecules to form centrosymmetric dimers. Weak π–π stacking inter­actions, with centroid–centroid distances of 3.779 (2) and 3.826 (2) Å, supply additional stabilization. The crystal packing also exhibits short inter­molecular contacts between the nitro groups and Br atoms [Br⋯O = 3.114 (2) Å].

## Related literature

For the crystal structure of the chloro analog of the title compound, see: Wang *et al.* (2009 ▶). For general background on the pharmacological activities of chalcones, see: Corréa *et al.* (2001 ▶); Jasinski *et al*. (2009 ▶); Nielsen *et al.* (1998 ▶); Vogel *et al.* (2008 ▶). For the synthetic details, see: Erhardt *et al.* (1985 ▶); Kranz *et al.* (1980 ▶).
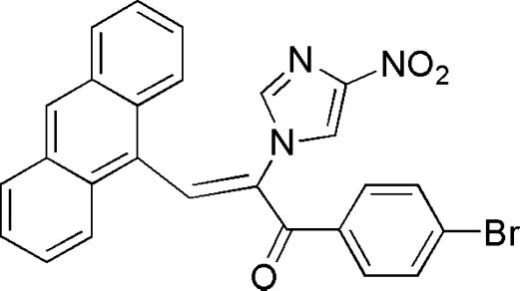


## Experimental

### 

#### Crystal data

C_26_H_16_BrN_3_O_3_
*M* *_r_* = 498.33
Triclinic, 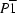

*a* = 8.1438 (11) Å
*b* = 11.0916 (14) Å
*c* = 12.7979 (17) Å
α = 78.146 (2)°
β = 86.193 (2)°
γ = 70.768 (2)°
*V* = 1068.2 (2) Å^3^
*Z* = 2
Mo *K*α radiation
μ = 1.96 mm^−1^
*T* = 292 K
0.13 × 0.12 × 0.10 mm

#### Data collection

Bruker SMART APEX CCD area-detector diffractometer
Absorption correction: multi-scan (*SADABS*; Sheldrick, 1996 ▶) *T*_min_ = 0.775, *T*_max_ = 0.828
6422 measured reflections
4315 independent reflections
3095 reflections with *I* > 2σ(*I*)
*R*_int_ = 0.019

#### Refinement

*R*[*F* ^2^ > 2σ(*F* ^2^)] = 0.044
*wR*(*F* ^2^) = 0.116
*S* = 1.02
4315 reflections
298 parameters
H-atom parameters constrained
Δρ_max_ = 0.56 e Å^−3^
Δρ_min_ = −0.67 e Å^−3^

### 

Data collection: *SMART* (Bruker, 2001 ▶); cell refinement: *SAINT-Plus* (Bruker, 2001 ▶); data reduction: *SAINT-Plus*; program(s) used to solve structure: *SHELXS97* (Sheldrick, 2008 ▶); program(s) used to refine structure: *SHELXL97* (Sheldrick, 2008 ▶); molecular graphics: *PLATON* (Spek, 2009 ▶); software used to prepare material for publication: *PLATON*.

## Supplementary Material

Crystal structure: contains datablocks global, I. DOI: 10.1107/S1600536809018352/lh2825sup1.cif

Structure factors: contains datablocks I. DOI: 10.1107/S1600536809018352/lh2825Isup2.hkl

Additional supplementary materials:  crystallographic information; 3D view; checkCIF report

## Figures and Tables

**Table 1:** Hydrogen-bond geometry (Å, °)

*D*—H⋯*A*	*D*—H	H⋯*A*	*D*⋯*A*	*D*—H⋯*A*
C23—H23⋯O3^i^	0.93	2.56	3.303 (4)	137

Symmetry code: (i) 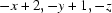.

**Fig. 1.**
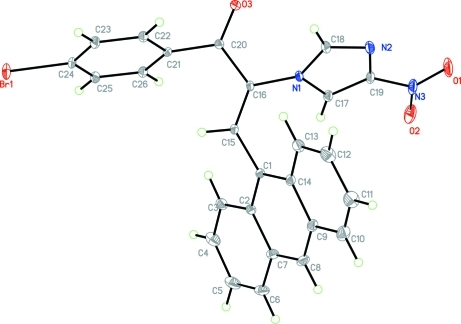
The molecular structure of (I), showing the atom-numbering scheme. Displacement ellipsoids are drawn at the 30% probability level.

**Fig. 2.**
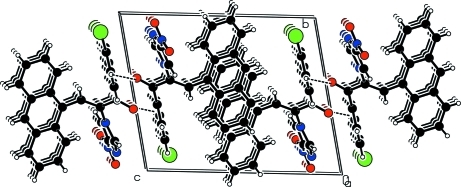
Part of the crystal structure of (I). Hydrogen bonds are shown as dashed lines.

## supplementary crystallographic information

### Comment

Chalcones and their derivatives have been reported responsible for a variety of pharmacological activities, including antibacterial, antifungal, anti-leishmanial, antimalarial, analgesic, anti-inflammatory and chemopreventive ones (Corréa *et al.*, 2001; Jasinski *et al.*, 2009; Simon *et al.*, 1998; Vogel *et al.*, 2008). Due to these varied applications, we have synthesized the title compound and report its crystal structure.

In the molecular structure of the title compound (I) (Fig. 1), the dihedral angle between the anthracene unit and imidazole ring is 63.79 (2) ° and that between the imidazole ring and benzene ring is 14.67 (2) °. In the crystal structure, weak intermolecular C—H···O hydrogen bonds link molecules to form centrosymmetric dimers (Fig. 2). Weak π–π staking interactions, with centroid to centroid distances of 3.779 (2) and 3.826 (2)Å supply additional stabilization.

### Experimental

Compound (I) was synthesized according to the procedure of Erhardt *et al.* (1985); Kranz  *et al.* (1980). A crystal suitable for X-ray analysis was grown from a chloroform and acetone solution of (I) by slow evaporation at room temperature.

### Refinement

H ydrogen atoms were placed in idealized positions with C—H = 0.93Å and *U*_iso_(H) = 1.2*U*_eq_(C).

### Crystal data

**Table:**

C_26_H_16_BrN_3_O_3_	*Z* = 2
*M**_r_* = 498.33	*F*(000) = 504
Triclinic, *P*1	*D*_x_ = 1.549 Mg m^−^^3^
Hall symbol: -P 1	Mo *K*α radiation, λ = 0.71073 Å
*a* = 8.1438 (11) Å	Cell parameters from 2344 reflections
*b* = 11.0916 (14) Å	θ = 2.3–26.9°
*c* = 12.7979 (17) Å	µ = 1.96 mm^−^^1^
α = 78.146 (2)°	*T* = 292 K
β = 86.193 (2)°	Block, orange
γ = 70.768 (2)°	0.13 × 0.12 × 0.10 mm
*V* = 1068.2 (2) Å^3^	

### Data collection

**Table:**

Bruker SMART APEX CCD area-detector diffractometer	4315 independent reflections
Radiation source: fine focus sealed Siemens Mo tube	3095 reflections with *I* > 2σ(*I*)
graphite	*R*_int_ = 0.019
0.3° wide ω exposures scans	θ_max_ = 26.5°, θ_min_ = 2.7°
Absorption correction: multi-scan (*SADABS*; Sheldrick, 1996)	*h* = −8→10
*T*_min_ = 0.775, *T*_max_ = 0.828	*k* = −13→13
6422 measured reflections	*l* = −16→15

### Refinement

**Table:**

Refinement on *F*^2^	Secondary atom site location: difference Fourier map
Least-squares matrix: full	Hydrogen site location: inferred from neighbouring sites
*R*[*F*^2^ > 2σ(*F*^2^)] = 0.044	H-atom parameters constrained
*wR*(*F*^2^) = 0.116	*w* = 1/[σ^2^(*F*_o_^2^) + (0.0524*P*)^2^ + 0.6665*P*] where *P* = (*F*_o_^2^ + 2*F*_c_^2^)/3
*S* = 1.02	(Δ/σ)_max_ = 0.008
4315 reflections	Δρ_max_ = 0.56 e Å^−^^3^
298 parameters	Δρ_min_ = −0.67 e Å^−^^3^
0 restraints	Extinction correction: *SHELXL97* (Sheldrick, 2008), Fc^*^=kFc[1+0.001xFc^2^λ^3^/sin(2θ)]^-1/4^
Primary atom site location: structure-invariant direct methods	Extinction coefficient: 0.0078 (11)

### Special details

**Table:**

Geometry. All e.s.d.'s (except the e.s.d. in the dihedral angle between two l.s. planes) are estimated using the full covariance matrix. The cell e.s.d.'s are taken into account individually in the estimation of e.s.d.'s in distances, angles and torsion angles; correlations between e.s.d.'s in cell parameters are only used when they are defined by crystal symmetry. An approximate (isotropic) treatment of cell e.s.d.'s is used for estimating e.s.d.'s involving l.s. planes.
Refinement. Refinement of *F*^2^ against ALL reflections. The weighted *R*-factor *wR* and goodness of fit *S* are based on *F*^2^, conventional *R*-factors *R* are based on *F*, with *F* set to zero for negative *F*^2^. The threshold expression of *F*^2^ > σ(*F*^2^) is used only for calculating *R*-factors(gt) *etc*. and is not relevant to the choice of reflections for refinement. *R*-factors based on *F*^2^ are statistically about twice as large as those based on *F*, and *R*- factors based on ALL data will be even larger.

### Fractional atomic coordinates and isotropic or equivalent isotropic displacement parameters (Å^2^)

**Table:**

	*x*	*y*	*z*	*U*_iso_*/*U*_eq_	
Br1	0.93749 (6)	0.89457 (4)	0.12755 (3)	0.06983 (18)	
C1	0.4551 (4)	0.4031 (3)	0.3854 (2)	0.0356 (6)	
C2	0.3193 (4)	0.4914 (3)	0.4320 (2)	0.0406 (7)	
C3	0.2357 (4)	0.6217 (3)	0.3799 (3)	0.0496 (8)	
H3	0.2699	0.6506	0.3111	0.060*	
C4	0.1080 (5)	0.7050 (4)	0.4276 (4)	0.0691 (11)	
H4	0.0565	0.7902	0.3917	0.083*	
C5	0.0521 (5)	0.6637 (5)	0.5312 (4)	0.0740 (13)	
H5	−0.0342	0.7224	0.5639	0.089*	
C6	0.1223 (5)	0.5405 (5)	0.5832 (3)	0.0647 (11)	
H6	0.0819	0.5144	0.6509	0.078*	
C7	0.2583 (4)	0.4483 (4)	0.5363 (2)	0.0510 (9)	
C8	0.3318 (5)	0.3209 (4)	0.5877 (3)	0.0570 (10)	
H8	0.2893	0.2930	0.6543	0.068*	
C9	0.4663 (5)	0.2335 (4)	0.5435 (3)	0.0545 (9)	
C10	0.5444 (7)	0.1041 (4)	0.5990 (3)	0.0804 (14)	
H10	0.5007	0.0765	0.6653	0.096*	
C11	0.6795 (8)	0.0206 (5)	0.5582 (4)	0.0943 (17)	
H11	0.7273	−0.0642	0.5957	0.113*	
C12	0.7504 (7)	0.0613 (4)	0.4575 (4)	0.0792 (13)	
H12	0.8461	0.0034	0.4305	0.095*	
C13	0.6790 (5)	0.1839 (3)	0.4007 (3)	0.0538 (9)	
H13	0.7270	0.2092	0.3353	0.065*	
C14	0.5326 (4)	0.2738 (3)	0.4394 (2)	0.0415 (7)	
C15	0.5261 (4)	0.4522 (3)	0.2830 (2)	0.0327 (6)	
H15	0.5626	0.5235	0.2820	0.039*	
C16	0.5458 (4)	0.4098 (2)	0.1916 (2)	0.0308 (6)	
C17	0.3147 (4)	0.3086 (3)	0.1964 (2)	0.0391 (7)	
H17	0.2215	0.3730	0.2185	0.047*	
C18	0.5712 (5)	0.1936 (3)	0.1475 (3)	0.0456 (7)	
H18	0.6886	0.1698	0.1296	0.055*	
C19	0.3157 (5)	0.1937 (3)	0.1754 (2)	0.0448 (8)	
C20	0.6441 (4)	0.4580 (3)	0.0995 (2)	0.0322 (6)	
C21	0.7038 (4)	0.5703 (3)	0.1060 (2)	0.0332 (6)	
C22	0.8822 (4)	0.5456 (3)	0.1056 (2)	0.0422 (7)	
H22	0.9569	0.4628	0.1014	0.051*	
C23	0.9499 (4)	0.6436 (3)	0.1113 (3)	0.0490 (8)	
H23	1.0696	0.6267	0.1122	0.059*	
C24	0.8383 (4)	0.7651 (3)	0.1157 (2)	0.0438 (7)	
C25	0.6602 (4)	0.7937 (3)	0.1138 (2)	0.0426 (7)	
H25	0.5861	0.8775	0.1155	0.051*	
C26	0.5943 (4)	0.6947 (3)	0.1094 (2)	0.0389 (7)	
H26	0.4746	0.7121	0.1087	0.047*	
N1	0.4811 (3)	0.3085 (2)	0.17774 (18)	0.0342 (5)	
N2	0.4740 (4)	0.1210 (2)	0.1464 (2)	0.0515 (7)	
N3	0.1680 (5)	0.1482 (3)	0.1818 (3)	0.0663 (9)	
O1	0.1909 (5)	0.0414 (3)	0.1602 (3)	0.1002 (11)	
O2	0.0289 (5)	0.2194 (4)	0.2088 (3)	0.0920 (10)	
O3	0.6822 (3)	0.4034 (2)	0.02424 (17)	0.0464 (5)	

### Atomic displacement parameters (Å^2^)

**Table:**

	*U*^11^	*U*^22^	*U*^33^	*U*^12^	*U*^13^	*U*^23^
Br1	0.0870 (3)	0.0579 (2)	0.0872 (3)	−0.0537 (2)	0.0032 (2)	−0.01466 (19)
C1	0.0423 (17)	0.0457 (16)	0.0284 (14)	−0.0269 (14)	0.0004 (12)	−0.0071 (12)
C2	0.0426 (18)	0.0551 (19)	0.0351 (16)	−0.0264 (15)	0.0010 (13)	−0.0159 (14)
C3	0.048 (2)	0.056 (2)	0.0514 (19)	−0.0203 (17)	0.0012 (15)	−0.0194 (16)
C4	0.058 (2)	0.073 (3)	0.079 (3)	−0.012 (2)	−0.004 (2)	−0.036 (2)
C5	0.049 (2)	0.108 (4)	0.079 (3)	−0.021 (2)	0.009 (2)	−0.057 (3)
C6	0.047 (2)	0.119 (4)	0.047 (2)	−0.040 (2)	0.0134 (17)	−0.038 (2)
C7	0.0468 (19)	0.089 (3)	0.0349 (17)	−0.0403 (19)	0.0018 (14)	−0.0203 (17)
C8	0.065 (2)	0.090 (3)	0.0297 (17)	−0.049 (2)	0.0024 (16)	−0.0047 (18)
C9	0.074 (3)	0.065 (2)	0.0363 (17)	−0.044 (2)	−0.0098 (17)	0.0023 (16)
C10	0.124 (4)	0.070 (3)	0.050 (2)	−0.049 (3)	−0.018 (2)	0.016 (2)
C11	0.156 (5)	0.054 (3)	0.061 (3)	−0.030 (3)	−0.026 (3)	0.016 (2)
C12	0.101 (3)	0.050 (2)	0.076 (3)	−0.008 (2)	−0.019 (2)	−0.009 (2)
C13	0.069 (2)	0.0462 (19)	0.0451 (19)	−0.0187 (18)	−0.0118 (17)	−0.0038 (15)
C14	0.0523 (19)	0.0446 (17)	0.0352 (16)	−0.0268 (15)	−0.0055 (14)	−0.0043 (13)
C15	0.0364 (16)	0.0331 (14)	0.0342 (15)	−0.0187 (12)	0.0007 (12)	−0.0065 (11)
C16	0.0360 (15)	0.0274 (13)	0.0345 (15)	−0.0171 (12)	−0.0003 (11)	−0.0066 (11)
C17	0.0398 (17)	0.0420 (16)	0.0412 (16)	−0.0219 (14)	−0.0027 (13)	−0.0052 (13)
C18	0.054 (2)	0.0340 (16)	0.0535 (19)	−0.0187 (14)	0.0031 (15)	−0.0123 (13)
C19	0.062 (2)	0.0461 (17)	0.0374 (16)	−0.0354 (17)	−0.0100 (15)	0.0011 (13)
C20	0.0317 (15)	0.0346 (14)	0.0327 (15)	−0.0129 (12)	0.0002 (12)	−0.0087 (12)
C21	0.0422 (17)	0.0358 (15)	0.0265 (14)	−0.0207 (13)	0.0045 (11)	−0.0049 (11)
C22	0.0423 (18)	0.0411 (16)	0.0498 (18)	−0.0197 (14)	0.0098 (14)	−0.0162 (14)
C23	0.0414 (18)	0.058 (2)	0.061 (2)	−0.0303 (16)	0.0103 (15)	−0.0203 (16)
C24	0.061 (2)	0.0409 (17)	0.0423 (17)	−0.0348 (16)	0.0083 (14)	−0.0084 (13)
C25	0.054 (2)	0.0329 (15)	0.0429 (17)	−0.0188 (14)	0.0001 (14)	−0.0040 (13)
C26	0.0395 (17)	0.0380 (16)	0.0430 (17)	−0.0178 (14)	0.0032 (13)	−0.0082 (13)
N1	0.0426 (14)	0.0311 (12)	0.0362 (12)	−0.0203 (11)	−0.0023 (10)	−0.0079 (10)
N2	0.075 (2)	0.0366 (14)	0.0513 (16)	−0.0297 (15)	−0.0045 (14)	−0.0068 (12)
N3	0.088 (3)	0.070 (2)	0.063 (2)	−0.061 (2)	−0.0171 (19)	0.0032 (16)
O1	0.133 (3)	0.089 (2)	0.121 (3)	−0.087 (2)	−0.009 (2)	−0.0254 (19)
O2	0.074 (2)	0.101 (2)	0.121 (3)	−0.063 (2)	−0.002 (2)	−0.007 (2)
O3	0.0553 (14)	0.0526 (13)	0.0437 (12)	−0.0285 (11)	0.0144 (10)	−0.0226 (10)

### Geometric parameters (Å, °)

**Table:**

Br1—C24	1.899 (3)	C15—C16	1.329 (4)
C1—C2	1.405 (4)	C15—H15	0.9300
C1—C14	1.410 (4)	C16—N1	1.434 (3)
C1—C15	1.471 (4)	C16—C20	1.487 (4)
C2—C3	1.419 (5)	C17—C19	1.353 (4)
C2—C7	1.436 (4)	C17—N1	1.359 (4)
C3—C4	1.349 (5)	C17—H17	0.9300
C3—H3	0.9300	C18—N2	1.305 (4)
C4—C5	1.408 (6)	C18—N1	1.365 (4)
C4—H4	0.9300	C18—H18	0.9300
C5—C6	1.341 (6)	C19—N2	1.351 (4)
C5—H5	0.9300	C19—N3	1.442 (4)
C6—C7	1.432 (5)	C20—O3	1.211 (3)
C6—H6	0.9300	C20—C21	1.497 (4)
C7—C8	1.379 (5)	C21—C26	1.382 (4)
C8—C9	1.379 (5)	C21—C22	1.387 (4)
C8—H8	0.9300	C22—C23	1.387 (4)
C9—C10	1.417 (5)	C22—H22	0.9300
C9—C14	1.444 (5)	C23—C24	1.365 (5)
C10—C11	1.338 (7)	C23—H23	0.9300
C10—H10	0.9300	C24—C25	1.380 (5)
C11—C12	1.426 (7)	C25—C26	1.384 (4)
C11—H11	0.9300	C25—H25	0.9300
C12—C13	1.358 (5)	C26—H26	0.9300
C12—H12	0.9300	N3—O1	1.222 (4)
C13—C14	1.415 (5)	N3—O2	1.224 (5)
C13—H13	0.9300		
			
C2—C1—C14	121.2 (3)	C16—C15—H15	115.2
C2—C1—C15	118.2 (3)	C1—C15—H15	115.2
C14—C1—C15	120.3 (3)	C15—C16—N1	121.6 (2)
C1—C2—C3	123.1 (3)	C15—C16—C20	122.8 (2)
C1—C2—C7	119.1 (3)	N1—C16—C20	115.5 (2)
C3—C2—C7	117.7 (3)	C19—C17—N1	104.3 (3)
C4—C3—C2	121.8 (4)	C19—C17—H17	127.8
C4—C3—H3	119.1	N1—C17—H17	127.8
C2—C3—H3	119.1	N2—C18—N1	112.3 (3)
C3—C4—C5	120.4 (4)	N2—C18—H18	123.9
C3—C4—H4	119.8	N1—C18—H18	123.9
C5—C4—H4	119.8	N2—C19—C17	112.9 (3)
C6—C5—C4	120.6 (4)	N2—C19—N3	121.2 (3)
C6—C5—H5	119.7	C17—C19—N3	125.9 (4)
C4—C5—H5	119.7	O3—C20—C16	120.7 (2)
C5—C6—C7	121.2 (4)	O3—C20—C21	121.2 (2)
C5—C6—H6	119.4	C16—C20—C21	118.0 (2)
C7—C6—H6	119.4	C26—C21—C22	119.1 (3)
C8—C7—C2	119.4 (3)	C26—C21—C20	124.6 (3)
C8—C7—C6	122.3 (3)	C22—C21—C20	116.3 (3)
C2—C7—C6	118.3 (3)	C23—C22—C21	120.5 (3)
C9—C8—C7	122.1 (3)	C23—C22—H22	119.8
C9—C8—H8	118.9	C21—C22—H22	119.8
C7—C8—H8	118.9	C24—C23—C22	119.0 (3)
C8—C9—C10	121.4 (4)	C24—C23—H23	120.5
C8—C9—C14	120.0 (3)	C22—C23—H23	120.5
C10—C9—C14	118.6 (4)	C23—C24—C25	122.0 (3)
C11—C10—C9	121.5 (4)	C23—C24—Br1	117.4 (2)
C11—C10—H10	119.2	C25—C24—Br1	120.6 (2)
C9—C10—H10	119.2	C26—C25—C24	118.4 (3)
C10—C11—C12	120.2 (4)	C26—C25—H25	120.8
C10—C11—H11	119.9	C24—C25—H25	120.8
C12—C11—H11	119.9	C21—C26—C25	121.0 (3)
C13—C12—C11	120.4 (4)	C21—C26—H26	119.5
C13—C12—H12	119.8	C25—C26—H26	119.5
C11—C12—H12	119.8	C17—N1—C18	106.8 (2)
C12—C13—C14	121.0 (4)	C17—N1—C16	125.3 (2)
C12—C13—H13	119.5	C18—N1—C16	128.0 (2)
C14—C13—H13	119.5	C18—N2—C19	103.7 (3)
C1—C14—C13	123.7 (3)	O1—N3—O2	124.9 (4)
C1—C14—C9	118.1 (3)	O1—N3—C19	117.7 (4)
C13—C14—C9	118.1 (3)	O2—N3—C19	117.4 (3)
C16—C15—C1	129.6 (2)		
			
C14—C1—C2—C3	−179.0 (3)	C1—C15—C16—C20	−170.6 (3)
C15—C1—C2—C3	6.7 (4)	N1—C17—C19—N2	1.0 (3)
C14—C1—C2—C7	−0.3 (4)	N1—C17—C19—N3	−178.9 (3)
C15—C1—C2—C7	−174.6 (2)	C15—C16—C20—O3	168.5 (3)
C1—C2—C3—C4	−178.6 (3)	N1—C16—C20—O3	−8.1 (4)
C7—C2—C3—C4	2.6 (5)	C15—C16—C20—C21	−7.7 (4)
C2—C3—C4—C5	−0.7 (5)	N1—C16—C20—C21	175.6 (2)
C3—C4—C5—C6	−1.4 (6)	O3—C20—C21—C26	118.6 (3)
C4—C5—C6—C7	1.5 (6)	C16—C20—C21—C26	−65.2 (4)
C1—C2—C7—C8	−1.2 (4)	O3—C20—C21—C22	−59.7 (4)
C3—C2—C7—C8	177.6 (3)	C16—C20—C21—C22	116.5 (3)
C1—C2—C7—C6	178.7 (3)	C26—C21—C22—C23	1.7 (4)
C3—C2—C7—C6	−2.5 (4)	C20—C21—C22—C23	−180.0 (3)
C5—C6—C7—C8	−179.5 (3)	C21—C22—C23—C24	−1.0 (5)
C5—C6—C7—C2	0.5 (5)	C22—C23—C24—C25	−0.4 (5)
C2—C7—C8—C9	1.9 (5)	C22—C23—C24—Br1	178.3 (2)
C6—C7—C8—C9	−178.0 (3)	C23—C24—C25—C26	1.2 (5)
C7—C8—C9—C10	177.8 (3)	Br1—C24—C25—C26	−177.4 (2)
C7—C8—C9—C14	−1.1 (5)	C22—C21—C26—C25	−0.9 (4)
C8—C9—C10—C11	−177.3 (4)	C20—C21—C26—C25	−179.1 (3)
C14—C9—C10—C11	1.7 (6)	C24—C25—C26—C21	−0.5 (4)
C9—C10—C11—C12	0.9 (7)	C19—C17—N1—C18	−0.3 (3)
C10—C11—C12—C13	−1.6 (7)	C19—C17—N1—C16	−178.5 (2)
C11—C12—C13—C14	−0.4 (6)	N2—C18—N1—C17	−0.5 (3)
C2—C1—C14—C13	−174.5 (3)	N2—C18—N1—C16	177.6 (3)
C15—C1—C14—C13	−0.3 (4)	C15—C16—N1—C17	53.4 (4)
C2—C1—C14—C9	1.1 (4)	C20—C16—N1—C17	−129.9 (3)
C15—C1—C14—C9	175.3 (3)	C15—C16—N1—C18	−124.4 (3)
C12—C13—C14—C1	178.5 (3)	C20—C16—N1—C18	52.4 (4)
C12—C13—C14—C9	3.0 (5)	N1—C18—N2—C19	1.0 (3)
C8—C9—C14—C1	−0.4 (4)	C17—C19—N2—C18	−1.3 (4)
C10—C9—C14—C1	−179.4 (3)	N3—C19—N2—C18	178.6 (3)
C8—C9—C14—C13	175.4 (3)	N2—C19—N3—O1	0.2 (5)
C10—C9—C14—C13	−3.6 (5)	C17—C19—N3—O1	−180.0 (3)
C2—C1—C15—C16	−126.2 (3)	N2—C19—N3—O2	179.8 (3)
C14—C1—C15—C16	59.4 (4)	C17—C19—N3—O2	−0.3 (5)
C1—C15—C16—N1	5.8 (5)		

### Hydrogen-bond geometry (Å, °)

**Table:**

*D*—H···*A*	*D*—H	H···*A*	*D*···*A*	*D*—H···*A*
C23—H23···O3^i^	0.93	2.56	3.303 (4)	137

Symmetry codes: (i) −*x*+2, −*y*+1, −*z*.
